# Cost-effectiveness analysis of the introduction of S-1 therapy for first-line metastatic breast cancer treatment in Japan: results from the randomized phase III SELECT BC trial

**DOI:** 10.1186/s12885-017-3774-7

**Published:** 2017-11-17

**Authors:** Takeru Shiroiwa, Takashi Fukuda, Kojiro Shimozuma, Mitsuko Mouri, Yasuhiro Hagiwara, Takuya Kawahara, Shozo Ohsumi, Yasuo Hozumi, Yoshiaki Sagara, Yasuo Ohashi, Hirofumi Mukai

**Affiliations:** 1 Department of Health and Welfare Services, National Institute of Public Health, 2-3-6 Minami, Wako, Saitama, 351-0197 Japan; 2 Department of Biomedical Sciences, College of Life Sciences, Ritsumeikan University, 1-1-1 Noji-higashi, Kusatsu, Shiga 525-8577 Japan; 3 Kanagawa Academy of Science and Technology (KAST), 3-2-1 Sakado, Takatsu-ku, Kawasaki, Kanagawa 213-0012 Japan; 4 Department of Biostatistics, School of Public Health, The University of Tokyo, 7-3-1 Hongo, Bunkyo-ku, Tokyo, 113-0033 Japan; 5 Biostatistics Division, Clinical Research Support Center, The University of Tokyo Hospital, 7-3-1 Hongo, Bunkyo-ku, Tokyo, 113-0033 Japan; 6 Department of Breast Oncology, National Hospital Organization Shikoku Cancer Center, 160 Kou, Minamiumemoto-machi, Matsuyama, Ehime 791-0280 Japan; 7 Department of Breast and Endocrine Surgery, University of Tsukuba Hospital, 2-1-1 Amakubo, Tsukuba, Ibaraki 305-8576 Japan; 8 Department of Breast Surgery, Ibaraki Prefectural Central Hospital, 6528 Koibuchi, Kasama, Ibaraki 309-1793 Japan; 9 Breast Surgery Department, Social Medical Corporation Hakuaikai Sagara Hospital, Matsubara-cho 3-31, Kagoshima, 892-0833 Japan; 10 Department of Integrated Science and Engineering, Chuo University, 1-13-27 Kasuga, Bunkyo-ku, Tokyo, 112-8551 Japan; 11 Division of Breast and Medical Oncology, National Cancer Center Hospital East, 6-5-1 Kashiwanoha, Kashiwa, Chiba 277-8577 Japan

**Keywords:** Cost-effectiveness analysis, Quality-adjusted life years, Breast neoplasms, Randomized controlled trial, S-1, Taxoids

## Abstract

**Background:**

This study evaluated the cost-effectiveness of replacing standard intravenous therapy (taxane) with oral S-1 therapy for first-line metastatic breast cancer treatment.

**Methods:**

This cost-effectiveness analysis was based on data from a randomized phase III trial (SELECT BC). As cost-effectiveness was a secondary endpoint of the SELECT BC trial, some of the randomized patients participated in an EQ-5D survey (*N* = 391) and health economic survey (*N* = 146). The EQ-5D responses, claims, and prescription data were collected for as long as possible until death. The expected quality-adjusted life years (QALY) obtained from each treatment were calculated using patient-level EQ-5D data, and the expected cost was calculated using patient-level claim data. The analysis was performed from the perspective of public healthcare payers.

**Results:**

The estimated EQ-5D least-square means and 95% CI up to 48 months were 0.764 (95% CI, 0.741–0.782) and 0.742 (95% CI, 0.720–0.764) in the S-1 and taxane arms, respectively. The expected QALY was 2.11 for the S-1 arm and 2.04 for the taxane arm, with expected costs of JPY 5.13 million (USD 46,600) and JPY 5.56 million (USD 50,500), respectively. These results show that S-1 is cost-saving. According to probabilistic sensitivity analysis, S-1 was dominant with a probability of 63%. When the willingness to pay (WTP) value was JPY 5 million (USD 45,500) per QALY, the probability of being cost-effective was 92%.

**Conclusions:**

Our results show that the introduction of oral S-1 therapy for metastatic breast cancer is highly likely to be cost-effective.

**Trial registration:**

UMIN CTR C000000416. Registered on May 10, 2006.

## Background

A number of novel anticancer drugs have been developed this decade, leading to a gradual improvement in the outcomes of cancer patients. However, the economic influence of these drugs on current public medical expenditures has become substantial due to their relatively high prices. Under these circumstances, and with the present budget constraints in healthcare, it is important to consider not only the safety and efficacy, but also the cost-effectiveness of anticancer drugs. In fact, many health technology assessment (HTA) organizations focus on new innovative anticancer drugs. For example, the National Institute for Health and Care Excellence (NICE) in the UK began evaluating all anticancer drugs in 2016 [1, 2] with the reform of cancer drugs fund. Some HTA agencies (e.g., NICE, the pan-Canadian Oncology Drug Review (pCODR) in Canada, and the Pharmaceutical Benefits Advisory Committee (PBAC) in Australia) concluded that certain chemotherapy regimens are not cost-effective and should not be recommended for routine use under the public healthcare system [3–5].

S-1 [6] (tegafur with gimeracil and oteracil, Teysuno®/TS-1®) is an oral fluoropyrimidine anticancer drug that does not require intravenous administration, unlike many other chemotherapy agents. Thus, patients receiving oral S-1 therapy do not need to bear long hours of intravenous administration and adverse events (e.g. phlebitis) associated with intravenous administration. In addition, a hospital visit is required to receive chemotherapy whenever intravenous anticancer drugs are administered. Therefore, S-1 may not only provide a convenient option for metastatic breast cancer (MBC) therapy, but may also improve the efficiency of treatment. S-1 has been approved in some Asian countries (Japan, Korea, Mainland China, Singapore, Taiwan, etc.) and European countries (UK, Germany, Sweden, etc.) for gastric cancer. However, Japan is the first country to avail S-1 to MBC patients.

SELECT BC [7] is a phase III, open-label, randomized controlled trial (RCT) that compared S-1 with taxanes (paclitaxel or docetaxel) for first-line MBC therapy. According to treatment algorithms (Hortobagyi [8] and NCCN guidelines [9]), patients irresponsive to endocrine therapy receive cytotoxic chemotherapy in standard cases. Taxanes are among the first-choice chemotherapy agents for MBC patients. However, the trial demonstrated non-inferiority of S-1 to taxane in overall survival (OS), with a median OS of 37.2 months in the taxane arm vs. 35.0 months in the S-1 arm (hazard ratio (HR) 1.05, 95% CI 0.86–1.27, *p* = 0.015), at a median follow-up of 34.6 months. The SELECT BC trial also evaluated cost-effectiveness as a secondary endpoint in addition to some clinical endpoints, including health-related quality of life (HRQOL).

In the SELECT BC trial, EuroQol 5-dimension (EQ-5D) measurements [10, 11] and claims (receipt) data collection for economic evaluation were also performed. These longitudinal patient-level EQ-5D and claims data can be used to calculate quality-adjusted life years (QALYs) and medical costs for evaluation of long-term cost-effectiveness. Such a trial-based [12, 13] cost-effectiveness analysis could improve the robustness of analysis and validity of internal comparison compared to a model-based approach [14] (e.g., using Markov model [15]). In this paper, we report on a cost-effectiveness analysis of the introduction of S-1 therapy to first-line MBC treatment using data from the SELECT BC trial.

## Methods

### Study design

In the SELECT BC trial, patients with HER2-negative, hormone-resistant MBC who were not previously treated with chemotherapy after diagnosis were randomized at a 1:1 ratio and allocated to the taxane arm (docetaxel 60–75 mg/m^2^ q3w, paclitaxel 80–100 mg/m^2^ q1w, or paclitaxel 175 mg/m^2^ q3w at the discretion of the treating physician) or S-1 arm (40–60 mg twice daily based on the patient’s body surface area, for 28 days on and 14 days off). Treatment continued until the disease progressed or more than four cycles of S-1 or six cycles of taxane were administered.

The enrollment period of the SELECT BC trial was from October 2006 to July 2010, and the trial involved 154 institutions in Japan. HRQOL was assessed using two instruments: the European Organization for Research and Treatment of Cancer Core Quality of Life Questionnaire C30 (EORTC QLQ-C30) [16] and EQ-5D. Not all 618 randomized patients responded to the HRQOL instruments; selection of HRQOL respondents was based on each institution. Some institutions were excluded in advance due to feasibility issues. Claims data were collected from a portion of the HRQOL population for the same reason. As institution was a prognostic factor for dynamic allocation, patient background factors were expected to be balanced in both arms.

The study was conducted in accordance with the Ethical Guidelines for Clinical Research of the Japanese Ministry of Health, Labour and Welfare and the Declaration of Helsinki. Written informed consent was obtained from each participant. Approval for the protocol and any modifications was obtained from an independent ethics committee of each participating institution. The SELECT BC trial was prospectively registered with the University Hospital Medical Information Network (UMIN) in Japan (protocol ID C000000416).

### EQ-5D assessment and claims data collection

EQ-5D is the most commonly used preference-based measure for assessing HRQOL [17, 18]. It can be used to calculate QALYs for the economic evaluation of healthcare technologies. We used the EQ-5D 3-level version, which comprises five items: “mobility,” “self-care,” “usual activities,” “pain/discomfort,” and “anxiety/depression,” at three levels of description. Responses can be converted to an EQ-5D score using a predetermined algorithm based on societal preferences of the general population [11].

In the SELECT BC trial, EQ-5D measurements were continued over a long period because measurements could be continued even when the disease progressed. Collection of monthly claims data was also continued to estimate treatment costs in the same manner. Patients responded to the Japanese version of the EQ-5D [11] at baseline and at 3, 6, and 12 months, and every 6 months thereafter until death or to the extent possible. In general, patients responded to the EQ-5D before the next cycle of chemotherapy was administered.

Claims data were created monthly by each institution for reimbursement of medical costs through public medical insurance in Japan. Claims data included all items of medical resources and drugs consumed in an institution, including those for adverse events. In addition, information on amounts and costs of each consumed item were included. We directly collected them from each institution, deleting patients’ personal information. However, claims data contained no information regarding pharmacy prescriptions. Accordingly, we also collected prescriptions from each institution. As claims data are not created by institutions when the patient’s monthly medical expenses were 0, we cannot distinguish whether the absence of claims data means no costs or missing data. Our data center contacted institutions to confirm whether no submission of claims data indicated no costs or missing data.

### Frameworks of cost-effectiveness analysis

We performed a cost-effectiveness analysis from the perspective of public healthcare payers. The time horizon was limited to 4 years, which is considered long enough to evaluate the values of healthcare technologies, given the quantity of collected claims data. The Japanese Breast Cancer Society clinical practice guidelines in 2013 recommended the use of anthracycline- or taxane-based regimens as first-line therapy for HER2 negative MBC patients. In 2015, the guidelines were revised to include S-1 in the recommended first-line therapies for HER2 negative patients [19] based on the results of the SELECT BC trial. Therefore, we selected taxanes as a comparator because taxanes are one of the standard therapies for first-line HER2 negative patients. The Japanese methodological guidelines for economic evaluation [20] recommend a 2% discount rate; therefore, cost and effectiveness was discounted by 2% per year, and the rate was changed from 0% to 4% as a sensitivity analysis in accordance with the guidelines. Unit costs were based on the Japanese fee schedule and drug tariff as of 2016, both of which are defined by the Ministry of Health, Labour and Welfare at an exchange rate of USD 1 = JPY 110 as of May 2016, as reported by the Bank of Japan.

The planned sample populations for the HRQOL analysis and cost analysis was approximately 300 and 150, respectively; these numbers were not based on a statistical calculation because HRQOL and cost-effectiveness in the SELECT BC trial were not the confirmatory endpoints. Collected responses were converted to EQ-5D index values [11].

Health outcomes of each intervention are evaluated in QALY. The expected QALY obtained from each treatment was calculated using patient-level data on survival and EQ-5D. Linear mixed models for repeated measures (MMRM) were applied to estimate EQ-5D scores. EQ-5D scores were adjusted by baseline score, treatment, time, and treatment-by-time interaction. Patient individual effect was also added to the model as a random effect. Responses with more than one missing items were treated as missing values, and they were analyzed based on “missing at random” assumption without any implementation. Estimates of the least-square means for EQ-5D score and 95% confidence intervals (CIs) were calculated by each visit and group. QALY between visits at t_i_ month and t_i + 1_ month was calculated by OS(t_i_) * 1/2(EQ5D(t_i_) + EQ5D(t_i + 1_)) * (t_i + 1_ - t_i_), using the estimated EQ-5D values. The expected cost (i.e., sum of costs from claims and prescription data) was calculated using patient-level survival and claims/prescription data by Lin’s method [21]; mean costs between visit (= total cost / number of observed patients) were multiplied by Kaplan-Meier estimator. If no claims data were collected, treatment costs for the corresponding month were treated as 0, unless claims data were no longer collected in future months. After the final claims data were received, subsequent data (until death) were censored.

Using estimates for expected costs and outcomes (QALY), incremental cost-effectiveness ratio (ICER) was calculated if superiority of EQ-5D values or OS (i.e., positive incremental effective value) was shown. However, it was clearly revealed that we could not expect superiority in HRQOL and OS. In such cases, if additional benefit could not be demonstrated, only the costs of both groups were compared based on the so-called “cost-minimization” approach in base-case analysis. The Bootstrap method (10,000 times resampling) was used for probabilistic sensitivity analysis, and a cost-effectiveness acceptability curve was created [22]. Unlike base-case analysis, the ICER may be calculated in each simulation [23].

As a scenario analysis, we adjusted drug costs by current drug prices as of May 2016. In Japan, drug prices generally decrease every 2 years based on the actual market price, with some exceptions. In addition, generics of taxane and S-1 are already in the market (breast cancer is not an indication for generics of S-1 yet, but S-1 for breast cancer will be off-patent in a few years). We also performed an analysis on generics by replacing taxane and S-1 with their average generic prices as of 2017, e.g., JPY 372.5 (USD 3.4) [S-1 25 mg capsule], JPY 14,798 (USD 134.5) [paclitaxel 100 mg vial], and JPY 29,802 (USD 270.9) [docetaxel 80 mg vial]. All analyses were performed with SAS® 9.4 and R 3.3.1.

## Results

### Patient population

Participants were 618 Japanese MBC patients randomly assigned to either the taxane (*N* = 309) or S-1 (*N* = 309) arm. A total of 175 and 208 patients in the taxane and S-1 arms, respectively, were included in the sample population for the HRQOL analysis. In the taxane arm, 96 patients received docetaxel and 79 received paclitaxel. Among patients subject to the cost analysis, 70 were allocated to the taxane (41 docetaxel and 29 paclitaxel) arm and 76 to the S-1 arm. Baseline characteristics of the patients were balanced between the two arms (Table 1) with similar distributions evident across the whole full analysis set (FAS) population.

**Table 1:** Patient demographics

	QOL population		Cost population		FAS population	
	Taxane	S-1	Taxane	S-1	Taxane	S-1
	*N* = 175	*N* = 208	*N* = 70	*N* = 76	*N* = 286	*N* = 306
Median age	57.0	59.0	58.0	59.0	58.5	59.0
Hormone receptor status						
ER-positive, PgR-positive, or both	127 (72.6)	149 (71.6)	50 (71.4)	54 (71.1)	212 (74.1)	223 (72.9)
ER-negative and PgR-negative	45 (25.7)	53 (25.5)	18 (25.7)	20 (26.3)	71 (24.8)	76 (24.8)
Unknown	3 (1.7)	6 (2.9)	2 (2.9)	2 (2.6)	3 (1.0)	7 (2.3)
HER2 status						
Negative	162 (92.6)	192 (92.3)	64 (91.4)	71 (93.4)	264 (92.3)	282 (92.2)
Unknown	13 (7.4)	16 (7.7)	6 (8.6)	5 (6.6)	22 (7.7)	24 (7.8)
Components of (neo)adjuvant treatment						
Oral fluoropyrimidine	26 (14.9)	22 (10.6)	9 (12.9)	10 (13.2)	39 (13.6)	35 (11.4)
Taxane	49 (28.0)	61 (29.3)	19 (27.1)	24 (31.6)	80 (28.0)	80 (26.1)
Endocrine therapy	100 (57.1)	111 (53.4)	44 (62.9)	45 (59.2)	170 (59.4)	169 (55.2)
Disease-free interval						
≤ 2 years	34 (19.4)	41 (19.7)	14 (20.0)	19 (25.0)	57 (19.9)	60 (19.6)
2–5 years	52 (29.7)	66 (31.7)	23 (32.9)	22 (28.9)	98 (34.3)	103 (33.6)
≥ 5 years	58 (33.1)	67 (32.2)	24 (34.3)	25 (32.9)	86 (30.0)	94 (30.7)
Unknown	0 (0.0)	0 (0.0)	0 (0.0)	0 (0.0)	0 (0.0)	2 (0.7)
No surgery	31 (17.7)	34 (16.3)	9 (12.9)	10 (13.2)	45 (15.7)	47 (15.4)
Liver metastasis						
Yes	61 (34.9)	78 (37.5)	20 (28.6)	27 (35.5)	96 (33.6)	103 (33.7)
No	114 (65.1)	130 (62.5)	50 (71.4)	49 (64.5)	190 (66.4)	203 (66.3)

### Completion rates of EQ-5D and the quantity of collected claims data

Longitudinal EQ-5D completion rates and the number of patients with collected claims data are shown in Table 2. The mean duration of EQ-5D measurements was 21 months for both groups. Completion rates at 3 months were 88.3% and 83.6% in the taxane and S-1 arms, respectively, and 71.8% and 77.6%, respectively, at 12 months. Although the percentage gradually declined with time, more than half of the patients completed the instrument up to 48 months. On the other hand, according to the record of the data center, the collection rate of claims data was roughly 100%. Thus, indications of no collected claims data should be interpreted as zero medical costs instead of missing data.

**Table 2:** Collection rate of EQ-5D and claims data

	QOL population		Cost population	
	Taxane	S-1	Taxane	S-1
	*N* = 175	*N* = 208	*N* = 70	*N* = 76
Baseline/Month 1	175/175 (100)	208/208 (100)	54	66
Month 3	151/171 (88.3)	168/201 (83.6)	70	70
Month 6	138/168 (82.1)	146/190 (76.8)	66	66
Month 12	107/149 (71.8)	132/170 (77.6)	49	56
Month 18	75/126 (59.5)	107/158 (67.7)	41	45
Month 24	68/117 (58.1)	93/137 (67.9)	38	45
Month 30	51/101 (50.5)	68/110 (61.8)	33	35
Month 36	45/90 (50.0)	47/84 (56.0)	32	27
Month 42	27/61 (44.3)	31/61 (50.8)	29	14
Month 48	18/39 (46.2)	21/37 (56.8)	18	15

### Cost-effectiveness of S-1 therapy

**Fig. 1:**
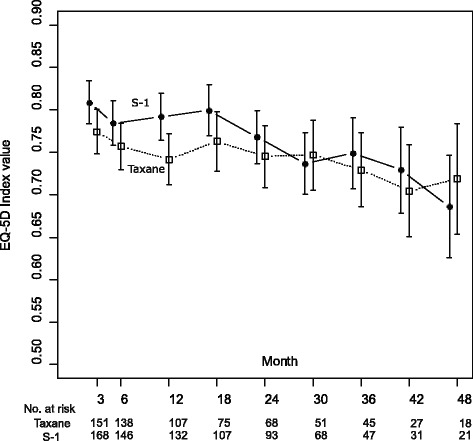
Longitudinal EQ-5D index. *Footnote of Fig. 1: This figure shows estimates of the least-square means for EQ-5D value and 95% confidence intervals. Black circle indicates values of S-1 group, and white square does those of taxane group

The longitudinal scores of the EQ-5D are shown in Fig. 1. The estimated EQ-5D least-square means and 95% CI up to 48 months were 0.764 (95% CI, 0.741–0.782) and 0.742 (95% CI, 0.720–0.764) in the S-1 and taxane arms, respectively (Appendix). EQ-5D values in the S-1 arm were not significantly larger than those in the taxane arm. The expected QALY was 2.11 for the S-1 arm and 2.04 for the taxane arm, while the expected costs were JPY 5.13 million (USD 46,600) and JPY 5.56 million (USD 50,500), respectively (Table 3). S-1 therapy was cost-saving by JPY 0.43 million (USD 3900) [SE: JPY 0.4 million], with increased QALY by 0.07 [SE: 0.09]. When OS data were extrapolated using Weibull regression analysis, the expected QALYs were approximately the same for S-1 (2.48) and taxane (2.50) arms.

**Table 3:** Results of cost-effectiveness analysis

Group	E (QALY)	IE (QALY)	C (JPY 1000)	IC (JPY 1000)
S-1	2.11	0.070	5307 [USD 47,000]	−424 [USD 3750]
Taxane	2.04		5731 [USD 50,700]	

*E* Effectiveness, *IE* Incremental effectiveness, *C* Cost, *IC* Incremental cost

According to the sensitivity analysis of the discount rate from 0% to 4%, incremental costs were not changed from PY 0.43 million (USD 3900). In the S-1 arm, outpatient cost was JPY 3.52 million (USD 32000), and inpatient cost was JPY 1.61 million (USD 14,600). In the taxane arm, outpatient cost was JPY 4.07 (USD 37,000), and inpatient cost was JPY 1.49 million (USD 13,500).

**Fig. 2:**
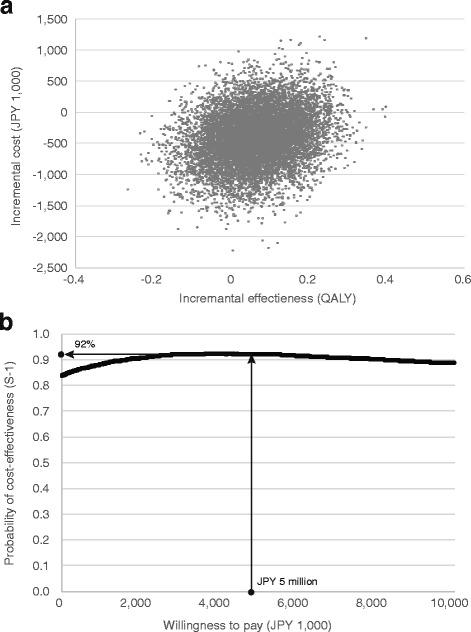
**a** Scatter plot on cost-effectiveness plane. **b** Cost-effectiveness acceptable curve. *Footnote of Figure2: These are results of probabilistic sensitivity analysis based on the bootstrap method. This scatter plot shows the joint distribution of incremental cost and effectiveness. Cost-effectiveness acceptable curve represents the relation between willingness to pay (or threshold) and the probability that S-1 is cost-effective

These results suggest that the S-1 arm obtained more QALYs at lower costs; i.e., that this option was dominant. According to probabilistic sensitivity analysis, the cost-effectiveness acceptability curve and scatter plot are presented in Fig. 2. The figure shows S-1 was dominant with a probability of 63% if the time horizon was limited to 4 years. When the willingness to pay (WTP) value was JPY 5 million (USD 45,500) per QALY [24], the probability of being cost-effective was 92%.

If drug prices were adjusted to current rates, the costs for both groups decreased to JPY 4.50 million (USD 40,900) in the S-1 arm and JPY 4.78 million (USD 43,400) in the taxane arm. In the S-1 and taxane arms, drug costs were JPY 1.14 million (USD 10,300) and JPY 1.48 million (USD 13,400), respectively.

The percentage of drug costs calculated by each of the four digits of the WHO-ATC code [25] was obtained (Table 4). The costs of L01 (antineoplastic agents) accounted roughly for more than 50% of drug costs. About 10–15% of drug costs were for M05 (drugs for treatment of bone diseases), into which mainly bisphosphonates for bone metastasis were classified. Analgesics (N02), endocrine therapy (L02), and antiemetics and antinauseants (A04) accounted for less than 10% of total drug costs.

**Table 4:** Percentage of drug costs classified by ATC

ATC code	ATC name	Taxane	S-1
L01	Antineoplastic agents	66%	59%
M05	Drugs for treatment of bone diseases	10%	16%
N02	Analgesics	9%	3%
L02	Endocrine therapy	3%	9%
A04	Antiemetics and antinauseants	3%	2%
V08	Contrast media	2%	2%
V09	Diagnostic raidopharmaceutical	1%	1%
A02	Drugs for acid and related disorders	1%	1%
	Others	5%	7%

Furthermore, when taxane and S-1 were replaced by generics, the cost of S-1 was JPY 4.16 million (USD 37,900), and taxane was JPY 4.39 million (USD 39,900). The cost difference between S-1 and taxane diminished if both taxane and S-1 were completely replaced by generics. When the price of generic S-1 was increased by more than 2.3 times, the cost of taxane was smaller than that of S-1.

## Discussion

We performed a cost-effectiveness analysis of oral S-1 therapy for MBC patients. The analysis was mainly based on information (survival, QOL, and treatment costs) collected from the Phase III randomized SELECT BC trial. Our results suggest that S-1 is cost-saving and the probability of being dominant (i.e., superior in both effectiveness and costs) is high compared with standard taxane therapy. A number of economic evaluations concluded that some anticancer drugs are either not cost-effective or have increased treatment costs even if they are cost-effective. However, our results revealed a high probability that S-1 therapy is cost-saving or dominant with high probability. Considering these results, S-1 may become one of the standard therapies used to treat MBC patients.

This study used Japanese unit costs (e.g., acquisition costs and drug prices) to estimate expected costs of chemotherapy, and our results cannot be simply extrapolated to other countries. However, in Europe, although S-1 has not yet been approved for MBC, the introduction of S-1 therapy for MBC patients may have larger economic effects because the difference in drug costs between the S-1 and taxane group was larger in both the UK and Germany than Japan. According to the British National Formulary (BNF) and Rote Liste, a 20 mg capsule of S-1 is JPY 564.7 (USD 5.1) in Japan, GBP 2.96 (USD 3.7, GBP 1 = USD 1.26) in UK and EUR 6.01 (USD 6.5, 1 EUR = USD 1.07) in Germany. For Docetaxel (Taxotel®), an 80 mg vial is JPY 52,835 (USD 480) in Japan, GBP 504.27 (USD 635) and EUR 783.17 (USD 838) in Germany. A 100 mg vial of Paclitaxel is JPY 22,071 (USD 201) in Japan, GBP 200.35 (USD 252) in UK and EUR 400.57 (USD 428.6) [the lowest price] in Germany. If the drug prices of S-1 and taxane in the UK were applied, the cost would be GBP 6200 (USD 7810) for S-1 and GBP 9310 (USD 11,700) for taxane. Similarly, drug cost as calculated by German pricing was EUR 11,900 (USD 12,700) for S-1 and EUR 16200 (USD 17,300) for taxane. Differences in drug costs between groups in the UK and Germany were larger than those in Japan, because the list price of taxane in the UK and Germany is higher than Japan; conversely, the cost of S-1 is similar or lower.

Our previous study examined longer-term (60 months) EQ-5D index values [26] and reported that the values were higher in the S-1 arm than the taxane arm when the analysis was limited to the first 12 months during progression-free survival (PFS). However, the values did not differ between arms when observations were continued up to 60 months. In the present evaluation, a 48-month analysis was performed to conform to the time horizon of cost-effectiveness analysis, although the above descriptions are also applied to the results of the EQ-5D in this analysis. This suggests that EQ-5D values of S-1 might be higher than those of taxane when patients receive chemotherapy. However, the difference was not statistically significant due to variation in EQ-5D values after chemotherapy, which are longer and more influential toward the results. In fact, the scores of EORTC QLQ-C30 were higher in the S-1 arm than in the taxane arm during 12 months from randomization for global health state (by 4.5; *p* = 0.039), as well as for all five functional domains including physical functioning, role functioning, emotional functioning, cognitive functioning, and social functioning [7].

In this study, the HRQOL and costs population comprised only a portion of the whole population. Only patients from contacted institutions completed the survey on HRQOL and costs. This design came about after considering the feasibility that some organization could not collect these data because of human resource restraints (e.g. lack of a clinical research coordinator at small institutions). Of course, while this design may have also caused potential selection bias of patients, institution was one of the adjusted factors for allocation in the SELECT BC trial. As shown in Table 1, the background factors of QOL and cost population were comparable to those of the whole FAS population. While the study design may be one limitation of the present investigation, we also feel that our results maintained high internal validity.

There are some limitations to claims data collection in randomized phase III trials. First, expenditures in a clinical trial and daily medical practice may not be the same. This may affect the generalizability of results [27]. However, we believe that the influence was similar in both arms. Second, in this trial, claims data were received from each institution with patient approval. As such, it was difficult to collect data if patients had received treatment from other clinics or hospitals, or changed their hospitals. For example, some patients might have been transferred to another institution to receive terminal care, but such data could not be collected. Although costs of terminal care may differ between the two groups, in many cases with cancer, most procedures are provided by experts; therefore, costs of cancer treatment provided by non-experts (i.e., other clinics and hospitals) can be regarded as unrelated medical costs [28]. Although there remains controversy about the handling of unrelated medical costs, the Japanese economic evaluation guideline [20] recommends that these costs should not be included in treatment costs. Lastly, claims data from pharmacies could not be collected for the same reason. Instead, we recorded prescribed drugs, which were then included in the costs of drugs. Calculating pharmacy fees in Japan is complicated (e.g., it depends on the type of pharmacy), and it is difficult to predict exact fees based only on the information provided by claims and prescription data. In this analysis, pharmacy fees were not included, although the standard pharmacy fee for 28 days of S-1 use ranges approximately from JPY 2000 to JPY 2500 based on a simple calculation. This was not reflected in our results.

The time horizon of our analysis was limited to 4 years. We believe this period is long enough to evaluate the cost-effectiveness of S-1 therapy. Normally, in an economic evaluation, a survival curve is estimated parametrically and extrapolated to obtain an estimated curve; the expected survival time or other measures are calculated using this curve. In the present analysis, the results were not changed even when the survival curve was extrapolated. Therefore, we used a more robust non-parametric Kaplan-Meier method without extrapolation.

The SELECT BC trial is one of the first oncology studies in Japan that collected EQ-5D and claims data continuously over a long period. The present analysis mainly used data from this trial, which enabled a robust analysis, and demonstrated that it is highly likely that oral S-1 therapy is cost-effective. In the area of outcomes research, attention is focused on real-world data (e.g., registry, claims database), although results sometimes have internal validity issues (even if external validity is high) when compared between two different treatment groups. We believe that trial- and real-world-based methods are complementary to each other, and even if studies based on real-world data increase due to improved availability of such data, the importance of trial-based analysis, such as the present study, should not be underestimated.

## Conclusions

Our results show that the introduction of oral S-1 therapy for metastatic breast cancer is cost-effective with a high probability. S-1 demonstrates potential for becoming a standard therapy for first-line metastatic breast cancer treatment in comparison with taxenes from the perspective of cost-effectiveness.

## Appendix

**Table 5:** Detailed results of EQ-5D analysis based on a mixed linear model

Effect	Group	Month	Estimate	Standard error	*p* -value
Intercept			0.3030	0.06016	<.0001
Baseline			0.4947	0.04474	<.0001
Group	Taxane		0.0328	0.07435	0.6591
	S-1		reference	.	.
Visit		3	0.1211	0.04914	0.0139
		6	0.0964	0.04925	0.0505
		12	0.1044	0.04935	0.0345
		18	0.1117	0.04956	0.0243
		24	0.0803	0.04975	0.1067
		30	0.0491	0.05033	0.3297
		36	0.0615	0.05138	0.2317
		42	0.0428	0.05324	0.4219
		48	−0.0014	0.05494	0.9793
		54	−0.0036	0.05813	0.9504
		60	reference	.	.
Group*visit	Taxane	3	−0.0671	0.07489	0.3701
		6	−0.0600	0.07500	0.4238
		12	−0.0831	0.07527	0.2699
		18	−0.0697	0.07578	0.3579
		24	−0.0553	0.07598	0.4665
		30	−0.0228	0.07681	0.7665
		36	−0.0526	0.07774	0.4991
		42	−0.0594	0.08048	0.4603
		48	−0.0005	0.08314	0.9951
		54	0.0294	0.08627	0.7334
		60	reference	.	.

## Abbreviations

BNF: British National Formulary
CI: Confidence interval
EORTC QLQ-C30: The European Organization for Research and Treatment of Cancer Core Quality of Life Questionnaire C30
EQ-5D: EuroQol 5-dimension
FAS: Full analysis set
HR: Hazard ratio
HRQOL: Health-related quality of life
HTA: Health technology assessment
ICER: Incremental cost-effectiveness ratio
MBC: Metastatic breast cancer
MMRM: Mixed models for repeated measures
NICE: National Institute for Health and Care Excellence
OS: Overall survival
PBAC: Pharmaceutical Benefits Advisory Committee
pCODR: The pan-Canadian Oncology Drug Review
PFS: Progression free survival
QALY: Quality-adjusted life year
RCT: Randomized controlled trial
UMIN: University Hospital Medical Information Network
WTP: Willingness to pay
